# Paying primary care practitioners to deliver alcohol brief interventions: the Scottish experience

**DOI:** 10.1186/1940-0640-7-S1-A16

**Published:** 2012-10-09

**Authors:** Jonathan Chick

**Affiliations:** 1 School of Health Sciences, Queen Margaret University, Lothian, Scotland, UK

## 

Health Improvement, Efficiency, Access to Services, and Treatment (HEAT) in the Scottish National Health Service (NHS) refers to a management system that includes a target to reduce health harms due to alcohol. The Government set NHS Scotland a HEAT target of delivering 149,449 alcohol brief interventions (ABIs) between 2008 and 2011. National guidance was offered on delivery models in a range of settings. Targeted rather than universal screening was recommended. Funds were made available to regions proportionate to the estimated number of harmful and hazardous drinkers in each region. In the region of Lothian, a “locally enhanced service contract” was agreed upon, the key components of which were adequate funding; centrally provisioned software that allowed easy data entry, payment, and auditing; and training and support for staff undertaking ABI screening and delivery. The software allowed a choice of screening tools or none. Minimum documentation was required. From October 2008 to mid 2010, 115 of the 126 practices in Lothian had contracted to provide this service, and 1236 staff were trained. For an adult population of approximately 800,000, it was estimated that 32% of women and 39% of men drink in excess of the recommended levels of 2-3 units daily for women and 3-4 units daily for men with two alcohol-free days per week—the highest of any Scottish health region. Therefore, the cumulative target number of ABIs to be achieved by the Lothian NHS by 31st March 2011 was 23,594, which was deemed likely to be reached. One outcome of the ABI initiative has been a national rise in referrals to secondary services, one that was anticipated by some extra funding. A fall in national consumption appears to be occurring, and, possibly, hospital admission rates as well, but the economic recession may be an additional explanation.

